# Supplementary material for: The Construction and Exploration of a Comprehensive MicroRNA Centered Regulatory Network in Foxtail Millet (Setaria italica L.)
Source: Front Plant Sci. 2022 May 6;13:848474. doi: 10.3389/fpls.2022.848474 (PMC9121102; doi:10.3389/fpls.2022.848474)

T=Seita.4G108600.1_Q=Sit−miR172a_S=1747

category=2_p=0.998227125404544

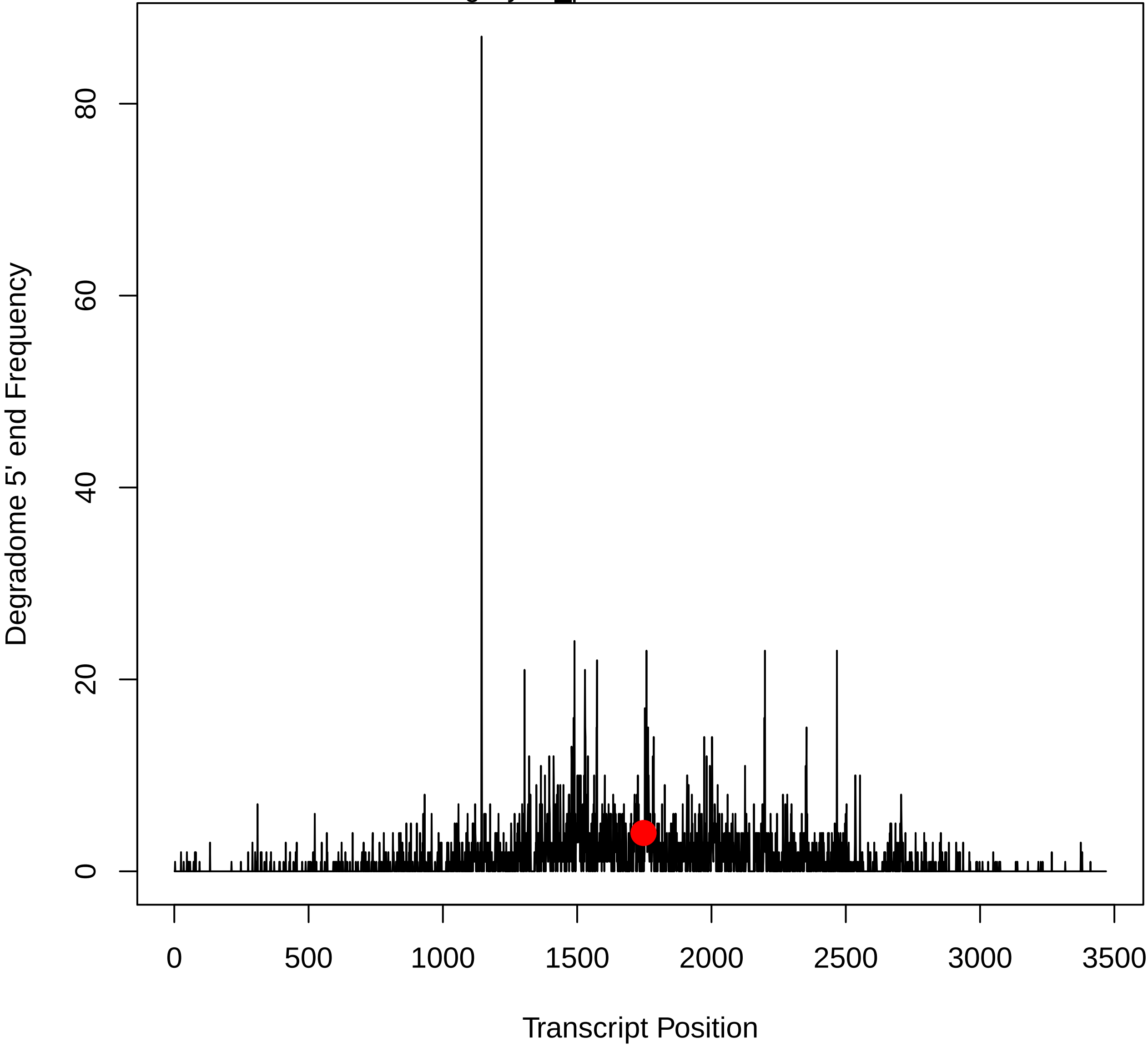

Supplement: Supplementary file 2 [file Data_Sheet_2.zip › Sit-miR172a_Seita.4G108600.1_1747_TPlot.pdf]
